# Outcomes and Prediction Models for Exclusive Prostate Bed Salvage Radiotherapy among Patients with Biochemical Recurrence after Radical Prostatectomy

**DOI:** 10.3390/cancers13112672

**Published:** 2021-05-28

**Authors:** Chi-Shin Tseng, Yu-Jen Wang, Chung-Hsin Chen, Shuo-Meng Wang, Kuo-How Huang, Po-Ming Chow, Yeong-Shiau Pu, Chao-Yuan Huang, Jason Chia-Hsien Cheng

**Affiliations:** 1 Graduate Institute of Clinical Medicine, National Taiwan University College of Medicine, Taipei 100233, Taiwan; clifford1987tcs@gmail.com; 2 Department of Urology, National Taiwan University College of Medicine and Hospital, Taipei 100225, Taiwan; mufasachen@gmail.com (C.-H.C.); dturo62smw@gmail.com (S.-M.W.); kuohowhuang@gmail.com (K.-H.H.); meow1812@gmail.com (P.-M.C.); yspu@ntu.edu.tw (Y.-S.P.); 3 Department of Medicine, National Taiwan University Hospital Jin-Shan Branch, New Taipei City 208204, Taiwan; 4 School of Medicine, College of Medicine, Fu Jen Catholic University, New Taipei City 242062, Taiwan; yujen.wang@gmail.com; 5 Department of Radiation Oncology and School of Medicine, Fu-Jen Catholic University Hospital and College of Medicine, New Taipei City 243089, Taiwan; 6 Division of Radiation Oncology, Department of Oncology, National Taiwan University College of Medicine and Hospital, Taipei 100229, Taiwan; 7 Graduate Institute of Oncology, National Taiwan University College of Medicine, Taipei 100233, Taiwan

**Keywords:** prostate cancer, radiotherapy, salvage, androgen-deprivation therapy, metastasis

## Abstract

**Simple Summary:**

Exclusive prostate bed salvage radiotherapy (SRT) without androgen deprivation therapy provides excellent treatment outcomes for selected patients with an increasing PSA after radical prostatectomy. We found that two risk factors, pre-SRT PSA and PSA-doubling time (PSA-DT), were shown to be predictive for clinical progression. According to the risk classification system proposed in the present study, men with a pre-SRT PSA < 0.45 ng/mL and PSA-DT > 8 months for post-prostatectomy biochemical recurrence (BCR) could be classified as “low risk” for recurrence or metastasis following SRT alone. Further prospective, multicenter studies are needed to validate these definitions. Nevertheless, individualized treatment decisions could be tailored based on these prediction models.

**Abstract:**

Background: The addition of androgen-deprivation therapy (ADT) or pelvic radiation to prostate bed salvage radiotherapy (SRT) has been debated for prostate cancer patients with biochemical recurrence (BCR) after radical prostatectomy. This study aimed to assess the outcomes and propose prediction models for exclusive prostate bed SRT. Methods: This is a prospective observational cohort study with patients who underwent SRT with a pre-SRT PSA < 1.5 ng/mL after radical prostatectomy. Patients were treated with 70-Gy SRT to the prostate bed exclusively. Kaplan–Meier survival analyses and Cox regression analyses were applied for depicting and predicting BCR-free survival, ADT-free survival, and metastasis-free survival (MFS). Regression-based coefficients were used to develop nomograms. Results: A total of 105 patients were included and 91 patients were eligible. The median follow-up period was 39 months. The 5-year BCR-free survival, ADT-free survival, and MFS were 37%, 50%, and 66%, respectively. Multivariable analysis showed that a pre-SRT PSA < 0.45 ng/mL was the only independent factor associated with longer BCR-free survival (*p* = 0.034), while a PSA-DT > 8 months had better ADT-free survival (*p* = 0.008). Patients with a PSA-DT > 8 months showed a 100% MFS and a 43% 5-year absolute benefit in MFS than a PSA-DT ≤ 8 months. All patients with a pre-SRT PSA < 0.45 ng/mL and PSA-DT > 8 months were free from subsequent ADT and any metastasis. Conclusions: In patients with a PSA < 0.45 ng/mL and PSA-DT > 8 months for post-prostatectomy BCR, prostate bed SRT provided excellent outcomes without the need for concomitant ADT or pelvic radiotherapy.

## 1. Introduction

A subsequently rising PSA develops in a substantial proportion of patients with localized prostate cancer after radical prostatectomy [1]. Although salvage radiotherapy (SRT) significantly delays long-term androgen-deprivation therapy (ADT) and reduces the risk of metastasis [2], less than half of the patients could maintain a 5-year PSA response after SRT [3,4].

According to the GETUG-AFU 16 trial with short-term ADT combined with SRT, the progression-free and metastasis-free survival improved significantly [5]. The multicenter Radiation Therapy Oncology Group (RTOG) 9601 trial showed a significantly better overall survival at 13 years in patients treated with a combination of SRT and high-dose antiandrogen therapy for two years [6]. However, the usage of ADT is associated with a decreased quality of life and might be harmful with overtreatment. In the subgroup analysis of RTOG 9601, patients treated at a pre-SRT PSA of 0.6 ng/mL or lower had no benefit in overall survival and was associated with increased non-cancer death from high-dose antiandrogen therapy [7].

Pelvic lymph nodes treatment by SRT in patients with no pathological node involvement has an increased complication rate and no significant benefit [8]. Although an ongoing trial has preliminarily reported an improved biochemical control by adding short-term ADT and SRT covering both the prostate bed and pelvic nodes [9], without a specific risk group stratification, whether a patient would benefit from either pelvic irradiation, systemic ADT, or both, remains unanswered.

Virtually none of the current trials focus on patients treated exclusively with SRT to the prostate bed, without the interference of ADT. In terms of the minimal extent of treatment, we investigate the most important prognosticators, the pre-SRT PSA level and PSA doubling time (PSA-DT), to select candidates for SRT. Several studies have proposed various pre-SRT PSA cut-off levels, but the comparative efficacy of early SRT at different PSA levels was controversial [3,10,11,12]. Therefore, we conducted a prospective observational trial to assess the outcomes and propose novel prediction models for the risks of biochemical recurrence and systemic metastasis following exclusive SRT to the prostate bed.

## 2. Material and Methods

This study was approved by the Institutional Review Board of National Taiwan University Hospital and was conducted according to the principles of the Declaration of Helsinki, including patient recruitment, waiver of informed consent due to the minimal risk of the study, and all the study methods.

### 2.1. Study Population

Between March 2007 and November 2019, 105 patients who received SRT alone after radical prostatectomy were enrolled. The surgery was undertaken with a curative intention by either laparoscopic or robotic-assisted method. Patients had a postoperative PSA concentration lower than 0.2 ng/mL. Patients who had neoadjuvant or adjuvant ADT were excluded. Prior to SRT, the PSA concentration began to rise without evidence of clinical metastatic disease. All patients had a life expectancy of 10 years or more, a Karnofsky Performance Score ≥ 80, no previous chemotherapy, and no history of pelvic radiation therapy.

### 2.2. Procedures

After radical prostatectomy, patients had regular visits at the urology clinic with PSA measurements every 3 to 6 months. PSA doubling time (PSA-DT) at relapse was calculated by dividing the natural log of 2 by the slope of the PSA. PSA velocity was calculated by dividing the increase in PSA (from nadir to pre-SRT PSA) by its time interval (months). When two consecutive PSA values above 0.2 ng/mL were shown, the timing and decision of SRT would be at the discretion of the physicians. Nevertheless, the patients were treated with pre-SRT PSA levels below 1.5 ng/mL, and the use of ADT was awaited. Patients with PSA relapse or reporting symptoms had a bone scan and CT/MRI for detecting recurrence or metastasis.

All patients received intensity-modulated RT to the prostate bed only with the clinical target volume following the EORTC guidelines for target volume definition in post-operative radiotherapy for prostate cancer [13]. The RT dose was 70 Gy by 10 MV photon radiation with 2 Gy per fraction per day in 35 fractions, 5 days a week for 7 weeks. Patients would not receive either concurrent or adjuvant ADT after SRT. Subsequently, the initiation of ADT would be determined by PSA recurrence or any metastasis.

### 2.3. Study Endpoints

The endpoints of the study included BCR-free survival, ADT-free survival, and MFS. BCR was defined as two consecutive PSA rises ≥ 0.2 ng/mL after SRT, and the first date was used for the survival analyses. The follow-up time was defined as the time from the initiation of SRT to the documented BCR or censoring at the date of the last follow-up. ADT-free survival was defined as the time from SRT to the ADT starting date. MFS was defined as the time from SRT to the documented metastasis. A metastasis event was defined as any radiological findings of metastatic sites other than the prostate bed. Data for adverse events potentially related to treatment were collected and graded according to the Common Terminology Criteria for Adverse Events version 4.

### 2.4. Statistical Analysis

Patients were stratified by a pre-SRT PSA of 0.45 ng/mL, in view of current recommendations for early SRT (given at a PSA ≤ 0.5 ng/mL) [5,10,12] and the mean PSA level of the study. The PSA-DT with a cutoff value of 8 months was analyzed, giving the assurance of no metastasis for patients with a longer PSA-DT (than 6 months) [5] and with the mean PSA-DT. The pre-SRT PSA and PSA-DT represented the two significant factors in predicting survival outcomes. The number of risk factors (0, 1, or 2) for stratification was used for further survival analysis. Statistical analyses were performed using commercial statistical software (SPSS, version 25.0; IBM Corp, SPSS, Inc., Chicago, IL, USA). Statistical analyses for nomogram construction were performed in R software (The R Foundation for Statistical Computing, Vienna, Austria).

## 3. Results

A total of 105 patients were enrolled in the study, and 91 eligible patients were analyzed. Table 1 summarizes the baseline characteristics of all patients, the early SRT group with a pre-SRT PSA < 0.45 ng/mL, and the late SRT group with a pre-SRT PSA of 0.45–1.5 ng/mL. Age, pathological T stage, adverse pathological features from radical prostatectomy, Gleason score, post-RP PSA nadir status, continence level after surgery, and the interval between surgery and SRT were similar between the two groups.

### 3.1. Survival Outcome Analyses

The median follow-up period for surviving patients was 38 months (IQR 19–53 months). Overall, the 5-year BCR-free survival, ADT-free survival, and MFS were 37%, 50%, and 66%, respectively. At data cutoff, 40 (44%) of the 91 patients had BCR after SRT, with two consecutive PSA values ≥ 0.2 ng/mL. Twenty-seven (30%) of the 91 patients received subsequent ADT for BCR after SRT. MFS events were diagnosed in 17 (19%) of the 91 patients. Four (4%) patients had pelvic lymph node recurrence, and 13 (14%) patients had distant metastasis outside the pelvis, including bone (10 patients), liver (1 patient), and lung (2 patients).

**Figure 1:**
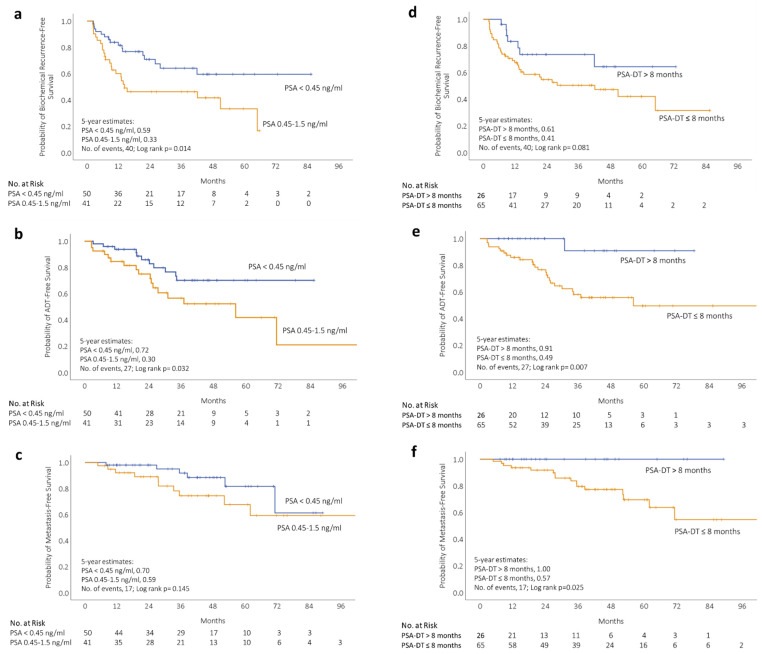
Kaplan–Meier survival curves depicting (**a**) biochemical recurrence-free, (**b**) ADT-free, and (**c**) metastasis-free survival after SRT in 91 patients stratified by pre-SRT PSA level into PSA < 0.45 ng/mL (blue) and PSA 0.45–1.5 ng/mL (orange). Kaplan–Meier survival curves depicting (**d**) biochemical recurrence-free, (**e**) ADT-free, and (**f**) metastasis-free survival after SRT in 91 patients stratified by PSA doubling time (PSA-DT) into PSA-DT > 8 months (blue) and PSA-DT ≤ 8 months (orange).

When patients were stratified according to the pre-SRT PSA, the 5-year BCR-free survival was 59% (95% CI 42–74) and 33% (95% CI 17–52) in the early and late SRT group, respectively (*p* = 0.014; Figure 1a); the 5-year ADT-free survival was 72% (95% CI 36–84) and 30% (95% CI 28–58) (*p* = 0.032; Figure 1b) and the 5-year MFS was not statistically different between the two groups (*p* = 0.145; Figure 1c).

PSA-DT was significantly prognostic for ADT-free survival (*p* = 0.007; Figure 1e) and MFS (*p* = 0.025; Figure 1f), but not BCR-free survival (*p* = 0.081; Figure 1d). Men with PSA-DT longer than 8 months had only 2 (8%) ADT-free survival events and no (0%) metastasis events, when compared to 25 (38%) ADT-free survival events and 17 (26%) metastasis-free survival events with PSA-DT ≤ 8 months.

### 3.2. Outcomes by the Risk Factors

**Figure 2:**
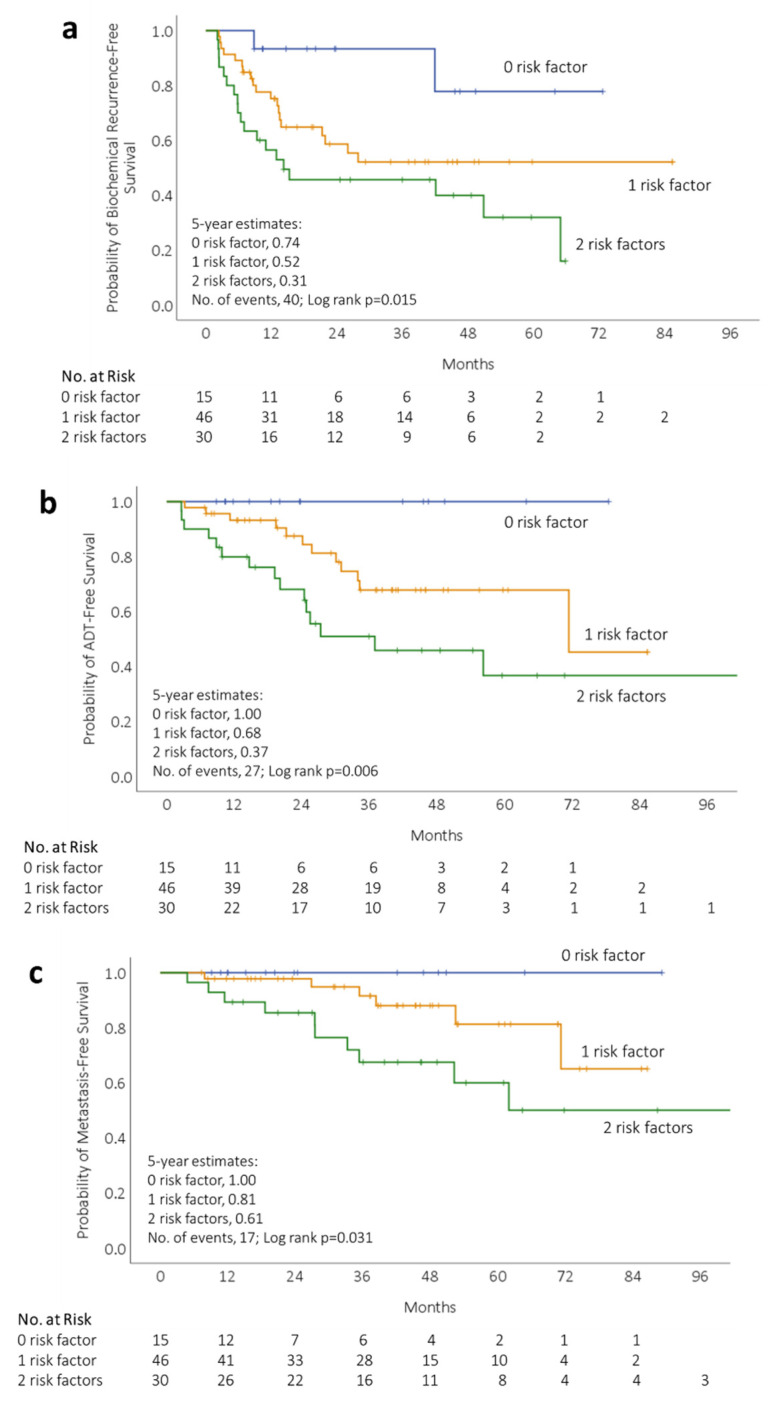
Kaplan–Meier survival curves depicting (**a**) biochemical recurrence-free, (**b**) ADT-free, and (**c**) metastasis-free survival after SRT in 91 patients stratified by risk factors into 0 risk factors (blue), 1 risk factor (orange), and 2 risk factors (green).

Patients stratified by the two risk factors (pre-SRT PSA and PSA-DT) exhibited different outcomes in BCR-free survival (*p* = 0.015; Figure 2a), ADT-free survival (*p* = 0.006, Figure 2b), and MFS (*p* = 0.031; Figure 2c). Patients with none of the risk factors demonstrated the most favorable outcomes, including no ADT-free survival event or MFS event. Patients with one risk factor, either pre-SRT PSA or PSA-DT, showed similar BCR rates of 45% and 40%, respectively (Table S1). Nevertheless, metastasis developed in 17% with one risk factor of PSA-DT ≤ 8 months and in 0% of patients with one risk factor of a pre-SRT PSA of 0.45–1.5 ng/mL, respectively.

### 3.3. Cox Regression Analyses

Univariable analysis showed that men with a pre-SRT PSA of 0.45–1.5 ng/mL had a significantly higher hazard in BCR-free survival (HR, 2.18; 95% CI, 1.16–4.10; *p* = 0.016) and ADT-free survival (HR, 2.29; 95% CI, 1.05–5.01; *p* = 0.038); men with PSA-DT ≤ 8 months had a significantly higher hazard in ADT-free survival (HR, 4.81; 95% CI, 1.14–20.34; *p* = 0.033) and had no metastasis-free survival event (Table 2). From the multivariate analysis, pre-SRT PSA was the only independent factor associated with BCR-free survival (HR, 2.03; CI, 1.05–3.90; *p* = 0.034; Table 2).

### 3.4. Nomograms Predicting the Outcome of SRT

**Figure 3:**
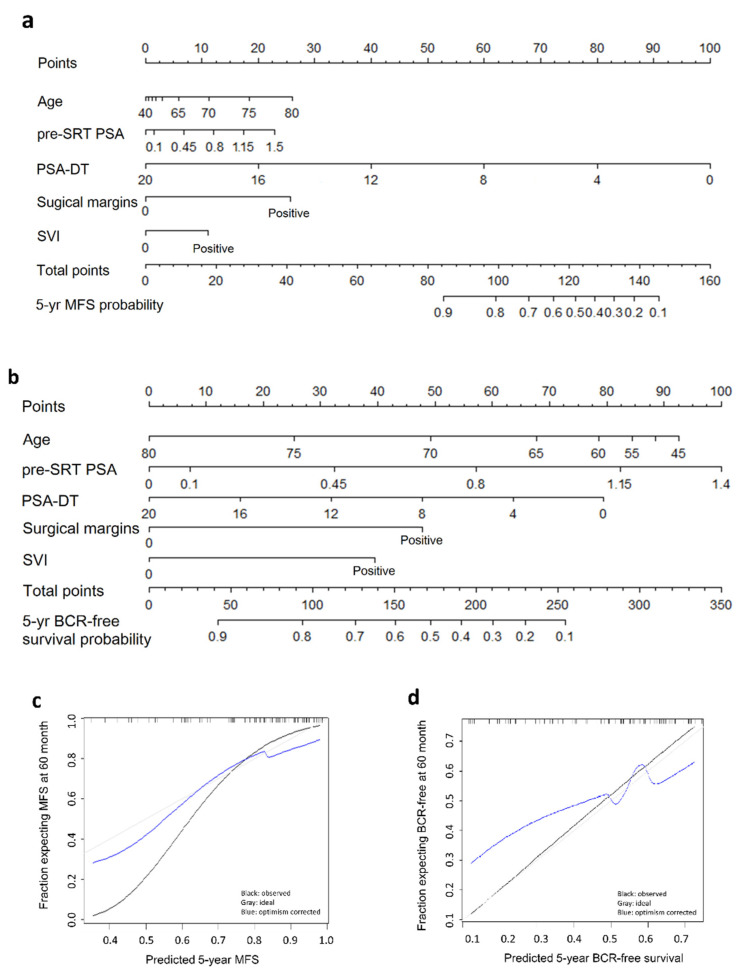
Nomogram to predict the 5-year (**a**) biochemical recurrence (BCR)-free probability and (**b**) metastasis-free (MSF) probability following salvage radiotherapy (SRT) for BCR after radical prostatectomy based on age at SRT, pre-SRT PSA level, PSA-doubling time (PSA-DT), surgical margins, and seminal vesicle invasion (SVI). Calibration of the nomogram for 5-year (**c**) BCR-free probability and (**d**) MSF probability.

Based on the coefficients of the Cox regression models that identified the predictors in either the BCR-free or metastasis-free model (age, pre-SRT PSA, PSA-DT, surgical margins, and SVI; Table 2), nomograms were developed to predict the 5-year BCR-free (Figure 3a) and metastasis-free probabilities (Figure 3b). These two nomograms visualized the important roles of pre-SRT PSA in predicting BCR and PSA-DT in predicting MFS. The nomograms were calibrated, with a correlation between the predicted and observed performance (Figure 3c,d).

### 3.5. Adverse Events of SRT

During the follow-up period, adverse events of Grades 1 to 3 were reported by six (6.6%) patients. No Grade 4 or 5 events were observed. Genitourinary adverse events of Grade 2 hematuria occurred in three patients. No aggravation of urinary incontinence was observed. Only one Grade 3 treatment-related colitis, one Grade 2 proctitis, and one Grade 1 dermatitis were reported.

## 4. Discussion

This prospective observational cohort study revealed the distinctive outcome of applying SRT to the prostate bed alone without the competing effect of concomitant ADT. Two significant factors, the pre-SRT PSA level and PSA-DT, could be used for predicting outcomes, deciding the best timing of SRT, and the necessity of additional treatment. Optimizing the efficacy of SRT is critical because long-term BCR-free control remains highly possible after solely using SRT.

Regarding pre-SRT PSA, in this study, patients undergoing late SRT at a PSA of 0.45–1.5 ng/mL had a 2-fold increase in BCR compared to early SRT with a PSA below 0.45 ng/mL. Abugharib et al. reported an increase in pre-SRT PSA (0.01–0.2, 0.2–0.5, >0.5 ng/mL) was associated with worse 10-year BCR-free survival (62%, 44%, and 27%) [10]. Another study reported that the advantage in 5-year BCR rates (42% versus 56%) was aligned with a lower pre-SRT PSA (≤0.5 than >0.5 ng/mL) [14]. A systematic review also suggested that better tumor control rates with SRT could be achieved with a lower pre-SRT PSA [15]. While the concept of early SRT has been widely adopted, we designated the cutoff value below 0.5 ng/mL to be well disposed to early SRT.

At the time of BCR after radical prostatectomy, the initial pathological grading by the Gleason score could not predict the outcome of SRT. PSA-DT < 3 months has been used as a surrogate for prostate cancer-specific mortality after surgery or radiation therapy in 8669 patients. Initiating ADT at the time of a BCR was recommended when the PSA-DT was less than 3 months to delay the imminent onset of metastatic bone disease [16]. Databases from the Center for Prostate Disease Research and Johns Hopkins University, including 656 men with BCR after prostatectomy, revealed that a PSA-DT < 7.5 months and Gleason score were independent risk factors for distant metastasis. Furthermore, the risk of metastasis increased from a PSA-DT of 6.01 to 7.50, 4.51 to 6.0, 3.01 to 4.50, and ≤3.0 months [17]. In the present study, a conclusion of a longer PSA-DT, 8 months, could be a protective value to maximize the sensitivity and specificity for MFS.

For patients with both risk factors, the incidences of BCR, ADT use, and distant metastasis were all significantly higher. Further investigations, such as SRT to additionally cover the pelvic nodes, are demanded to intensify the treatment. The RTOG 0534 trial was designed with three arms: SRT prostate bed alone, SRT to the prostate bed with 4–6 months of ADT, and SRT to the prostate bed and pelvic nodes with 4–6 months of ADT; the preliminarily reported values for the 5-year BCR-free survival are 71.1, 82.7, and 89.1%, respectively [9]. Although the trial enrolled only 17% of patients with a pathologic Gleason score of 8 or higher and 90% of patients had a pre-SRT PSA < 1.0 ng/mL, the combinational treatment might potentially become the standard practice for high-risk patients.

Patients at “high risk” of BCR after post-prostatectomy SRT in previous trials were selected by meeting any of the following criteria: PSA-DT < 6 months, PSA at SRT > 1 ng/mL, Gleason score > 7, positive surgical margin, and seminal vesicle involvement [3,4,18]. Another BCR risk stratification, based on a systemic review, defined “high-risk” as meeting one of the following risk criteria: PSA-DT < 1 year, pathological Gleason score of 8–10 from prostatectomy, the interval to biochemical failure after prostatectomy ≤ 18 months, or a high biopsy Gleason score of 8–10 [19]. According to these criteria, patients with either adverse pathological feature or Gleason score > 7, which failed to predict outcome in the present study, would be regarded as high risk, regardless of a long PSA-DT and low pre-SRT PSA. For instance, in GETUG AFU-16 trial, patients were at a favorable condition, including an undetectable PSA after radical prostatectomy and low pre-SRT PSA levels (median pre-SRT PSA was 0.3 ng/mL with an IQR of 0.2–0.5 ng/mL) [5]. As a matter of fact, the high-risk subgroup in that trial, which was mainly based on adverse pathological features or Gleason score, failed to demonstrate MFS benefits by adding short-term ADT to SRT. Therefore, many of the abovementioned variables and criteria should be re-evaluated in terms of clinical effectiveness and practicality.

In this study, patients with zero risk factors, reporting excellent outcomes following SRT alone, would derive minimal benefit from ADT. On the contrary, RTOG 9601 study showed a detrimental effect of the use of 2-year anti-androgen therapy which was associated with a greater than 3-fold increase in high-grade cardiac and neurogenic events and a 2-fold increase in other-cause mortality in patients undergoing SRT at PSA levels of 0.6 ng/mL or lower [7].

Nevertheless, SRT to the prostate bed rarely causes severe adverse events. In the present study, only five (5.5%) patients had Grade 2 or more adverse events relevant to SRT, including three (3.3%) cases with hematuria, one (1.1%) with colitis, and one (1.1%) with proctitis. The low toxicity rate could be explained by the precise and confined target of the SRT. In a retrospective study on 959 patients receiving either adjuvant radiotherapy or SRT, 78% of patients were treated at the prostate bed only, and 22% at the pelvis. In comparison, 4% and 0.4% had Grade 2 and Grade 3 late gastrointestinal toxicity, and 10% and 1% had Grade 2 and Grade 3 late genitourinary toxicity at 5 years, respectively [20].

The present study had several advantages, such as a contemporary cohort starting from March 2007, modern treatment modalities with minimally invasive surgery, exclusively intensity-modulated radiation therapy to the prostate bed, and a natural course of SRT without the interference of ADT. The limitations of the study included the small number of cases, short follow-up period, and no mature cancer-specific survival or overall survival results.

## 5. Conclusions

The stratification system, an integration of the two predictive factors, pre-SRT PSA and PSA-DT, is highly prognostic for BCR after SRT, freedom from ADT use, and distant metastasis. Further prospective studies, with more participants enrolled, would be required to confirm the prediction models among patients with biochemical recurrence after radical prostatectomy.

## Figures and Tables

**Table 1:** Clinical characteristics of patients undergoing salvage radiotherapy for recurrence after radical prostatectomy.

Characteristic	All Patients	Presalvage Radiotherapy PSA Levels		*p*-Value
		PSA < 0.45 ng/mL	PSA 0.45–1.5 ng/mL	
Patients, No.	91	50	41	
Age at radiotherapy, median (IQR)	66.0 (61.8–69.3)	67.4 (63.3–69.9)	65.1 (61.1–68.9)	0.112
Pathological T stage (%)				0.462 *
T2	34 (37%)	17 (34%)	17 (41.5%)	
T3	54 (59%)	31 (62%)	23 (56.1%)	
Unknown	3 (3%)	2 (4%)	1 (2.4%)	
Extra prostatic extension (%)	52 (57%)	30/50 (60%)	22/41 (53.6%)	0.664 *
Seminal vesicle invasion (%)	23 (35%)	10/50 (20%)	13/41 (31.7%)	0.164 *
Grade group (%)				0.124 *
3 + 3, 3 + 4	35 (38%)	20/50 (40%)	15/41 (36.6%)	
4 + 3	31 (34%))	13/50 (26%)	18/41 (43.9%)	
≥8	23 (25%)	16/50 (32%)	7/41 (17.1%)	
Unknown	3 (3%)	1/50 (2%)	1/41 (2.4%)	
Surgical margin (%)				0.573 *
Negative	27 (31%)	14/49 (28.6%)	13/38 (34.2%)	
Positive	60 (69%)	35/49 (71.4%)	25/38 (65.8%)	
PSA nadir after surgery (ng/mL)		0.03	0.033	0.876
PSA < 0.1 ng/mL	68 (76%)	42/49 (85.7%)	26/41 (63.4%)	
PSA ≥ 0.1 ng/mL	22 (24%)	7/49 (14.3%)	15/41 (36.6%)	
Continence (%)				0.058 *
0–1 pad/day	71 (96%)	43/45 (95.6%)	29/30 (96.7%)	
≥2 pad/day	3 (4%)	2/45 (4.4%)	1/30 (3.3%)	
PSA velocity (ng/mL/year), median (IQR)	0.137 (0.004–0.402)	0.110 (0.004–0.306)	0.173 (0.004–0.690)	0.127
Time from surgery to SRT (months), median (IQR)	18.0 (20.5–36.2)	17.9 (10.6–28.5)	18.0 (10.4–41.1)	0.922
Presalvage radiotherapy PSA levels, median (IQR)	0.41 (0.29–0.59)	0.29 (0.26–0.34)	0.59 (0.54–0.87)	<0.001

* Pearson Chi-Square.

**Table 2:** Univariable and multivariable analysis for biochemical recurrence (BCR)-free survival, androgen-deprivation therapy (ADT)-free survival, and metastasis-free survival for patients with a pre-salvage radiotherapy PSA below 1.5 ng/mL.

Analysis	BCR-Free Survival		ADT-Free Survival		Metastasis-Free Survival	
	(*n* = 91)	*p*-Value	(*n* = 91)	*p*-Value	(*n* = 91)	*p*-Value
	HR (95% CI)		HR (95% CI)		HR (95% CI)	
**Univariable analysis**						
Age at radiotherapy	0.97 (0.93–1.01)	0.154	0.95 (0.91–0.99)	0.033	1.02 (0.95–1.11)	0.588
Pathological T stage, T2 vs. T3 (ref)	0.74 (0.37–1.48)	0.394	0.46 (0.18–1.18)	0.105	0.66 (0.23–1.89)	0.437
EPE, negative vs. positive (ref)	0.83 (0.42–1.64)	0.593	0.50 (0.20–1.28)	0.148	0.59 (0.20–1.73)	0.34
SVI, negative vs. positive (ref)	0.53 (0.28–1.04)	0.063	0.50 (0.23–1.11)	0.089	0.50 (0.19–1.36)	0.175
Gleason score, 6–7 vs. 8–10 (ref)	0.87 (0.43–1.77)	0.707	0.52 (0.23–1.16)	0.11	0.55 (0.19–1.54)	0.253
Margin, negative vs. positive (ref)	0.62 (0.32–1.21)	0.16	0.76 (0.33–1.77)	0.525	0.40 (0.15–1.08)	0.079
Incontinence vs. Continence (ref)	1.32 (0.31–5.55)	0.705	1.83 (0.42–7.96)	0.419	1.91 (0.24–15.16)	0.541
PSA velocity						
<0.3 ng/mL/year	Reference	-	Reference	-	Reference	-
≥0.3 ng/mL/year	1.2 (0.64–2.25)	0.566	2.69 (1.12–6.42)	0.026	1.31 (0.48–3.58)	0.596
Time between surgery and recurrence						
<24 months	Reference	-	Reference	-	Reference	-
≥24 months	0.69 (0.37–1.30)	0.255	0.91 (0.40–2.08)	0.82	0.57 (0.21–1.49)	0.249
Presalvage Radiotherapy PSA						
PSA < 0.45 ng/mL	Reference	-	Reference	-	Reference	-
PSA 0.45–1.5 ng/mL	2.18 (1.16–4.10)	0.016	2.29 (1.05–5.01)	0.038	1.97 (0.71–5.45)	0.19
PSA doubling time at relapse						
>8 months	Reference	-	Reference	-	Reference	(0 event)
≤8 months	2.04 (0.90–4.61)	0.088	4.81 (1.14–20.34)	0.033	-	-
**Multivariable analysis**						
Age at radiotherapy	-	-	0.92 (0.86–0.99)	0.021	-	-
SVI, negative vs. positive (ref)	0.59 (0.30–1.18)	0.136	0.42 (0.16–1.07)	0.069	-	-
Margin, negative vs. positive (ref)	-	-	-	-	0.40 (0.15–1.09)	0.074
PSA velocity						
<0.3 ng/mL/year	-	-	-	-	-	-
≥0.3 ng/mL/year	-	-	1.49 (0.50–4.43)	0.475	-	-
Presalvage Radiotherapy PSA						
PSA < 0.45 ng/mL	Reference	-	Reference	-	Reference	-
PSA 0.45–1.5 ng/mL	2.03 (1.05–3.90)	0.034	1.78 (0.72–4.38)	0.212	2.26 (0.81–6.27)	0.117
PSA doubling time at relapse						
>8 months	Reference	-	Reference	-	Reference	(0 event)
≤8 months	1.96 (0.86–4.45)	0.11	11.21 (1.87–67.23)	0.008	-	-

## Data Availability

The data are publicly accessible to qualified investigators upon request.

## Supplementary Materials

The following are available online at https://www.mdpi.com/article/10.3390/cancers13112672/s1, Table S1. Biochemical recurrence (BCR), androgen-deprivation therapy (ADT), and metastasis event numbers according to different combinations of the risk factors: Pre-salvage radiotherapy (SRT) PSA and PSA-doubling time (PSA-DT).

## Abbreviations

ADT = androgen-deprivation therapy; BCR = biochemical recurrence; CI = confidence interval; EORTC = European Organization for Research and Treatment of Cancer; HR = hazard ratio; MFS = metastasis-free survival; PSA = prostate-specific antigen; PSA-DT = PSA doubling time; RT = radiation therapy; RTOG = Radiation Therapy Oncology Group; SRT = salvage radiotherapy; SVI = seminal vesicle invasion.

## References

[1] Abdollah F., Sood A., Sammon J.D., Hsu L., Beyer B., Moschini M., Gandaglia G., Rogers C.G., Haese A., Montorsi F. (2015). Long-term cancer control outcomes in patients with clinically high-risk prostate cancer treated with robot-assisted radical prostatectomy: Results from a multi-institutional study of 1100 patients. Eur. Urol..

[2] Boorjian S.A., Karnes R.J., Crispen P.L., Rangel L.J., Bergstralh E.J., Blute M.L. (2009). Radiation therapy after radical prostatectomy: Impact on metastasis and survival. J. Urol..

[3] Stephenson A.J., Scardino P.T., Kattan M.W., Pisansky T.M., Slawin K.M., Klein E.A., Anscher M.S., Michalski J.M., Sandler H.M., Lin D.W. (2007). Predicting the outcome of salvage radiation therapy for recurrent prostate cancer after radical prostatectomy. J. Clin. Oncol..

[4] Freedland S.J., Humphreys E.B., Mangold L.A., Eisenberger M., Dorey F.J., Walsh P.C., Partin A.W. (2005). Risk of prostate cancer-specific mortality following biochemical recurrence after radical prostatectomy. JAMA.

[5] Carrie C., Magné N., Burban-Provost P., Sargos P., Latorzeff I., Lagrange J.-L., Supiot S., Belkacemi Y., Peiffert D., Allouache N. (2019). Short-term androgen deprivation therapy combined with radiotherapy as salvage treatment after radical prostatectomy for prostate cancer (GETUG-AFU 16): A 112-month follow-up of a phase 3, randomised trial. Lancet Oncol..

[6] Shipley W.U., Seiferheld W., Lukka H.R., Major P.P., Heney N.M., Grignon D.J., Sartor O., Patel M.P., Bahary J.-P., Zietman A.L. (2017). Radiation with or without Antiandrogen Therapy in Recurrent Prostate Cancer. N. Engl. J. Med..

[7] Dess R.T., Sun Y., Jackson W.C., Jairath N.K., Kishan A.U., Wallington D.G., Mahal B.A., Stish B.J., Zumsteg Z.S., Den R.B. (2020). Association of Presalvage Radiotherapy PSA Levels After Prostatectomy with Outcomes of Long-term Antiandrogen Therapy in Men With Prostate Cancer. JAMA Oncol..

[8] Dirix P., Haustermans K., Junius S., Withers R., Oyen R., Van Poppel H. (2006). The role of whole pelvic radiotherapy in locally advanced prostate cancer. Radiother Oncol..

[9] Pollack A., Karrison T., Balogh A., Low D., Bruner D., Wefel J., Gomella L., Vigneault E., Michalski J., Angyalfi S. (2018). Short Term Androgen Deprivation Therapy Without or With Pelvic Lymph Node Treatment Added to Prostate Bed Only Salvage Radiotherapy: The NRG Oncology/RTOG 0534 SPPORT Trial. Int. J. Radiat. Oncol. Biol. Phys..

[10] Abugharib A., Jackson W.C., Tumati V., Dess R.T., Lee J.Y., Zhao S.G., Soliman M., Zumsteg Z.S., Mehra R., Feng F.Y. (2017). Very Early Salvage Radiotherapy Improves Distant Metastasis-Free Survival. J. Urol..

[11] Buskirk S.J., Pisansky T.M., Schild S.E., Macdonald O.K., Wehle M.J., Kozelsky T.F., Collie A.C., Ferrigni R.G., Myers R.P., Prussak K.A. (2006). Salvage radiotherapy for isolated prostate specific antigen increase after radical prostatectomy: Evaluation of prognostic factors and creation of a prognostic scoring system. J. Urol..

[12] Briganti A., Wiegel T., Joniau S., Cozzarini C., Bianchi M., Sun M., Tombal B., Haustermans K., Budiharto T., Hinkelbein W. (2012). Early salvage radiation therapy does not compromise cancer control in patients with pT3N0 prostate cancer after radical prostatectomy: Results of a match-controlled multi-institutional analysis. Eur. Urol..

[13] Poortmans P., Bossi A., Vandeputte K., Bosset M., Miralbell R., Maingon P., Boehmer D., Budiharto T., Symon Z., Bergh A.C.V.D. (2007). Guidelines for target volume definition in post-operative radiotherapy for prostate cancer, on behalf of the EORTC Radiation Oncology Group. Radiother Oncol..

[14] Stish B.J., Pisansky T.M., Harmsen W.S., Davis B.J., Tzou K.S., Choo R., Buskirk S.J. (2016). Improved Metastasis-Free and Survival Outcomes with Early Salvage Radiotherapy in Men With Detectable Prostate-Specific Antigen After Prostatectomy for Prostate Cancer. J. Clin. Oncol..

[15] Christopher R.K. (2012). The Timing of Salvage Radiotherapy After Radical Prostatectomy: A Systematic Review. Int. J. Radiat. Oncol. Biol. Phys..

[16] D’Amico A.V., Moul J.W., Carroll P.R., Sun L., Lubeck D., Chen M.-H. (2003). Surrogate end point for prostate cancer-specific mortality after radical prostatectomy or radiation therapy. J. Natl. Cancer Inst..

[17] Markowski M.C., Chen Y., Feng Z., Cullen J., Trock B.J., Suzman D., Antonarakis E.S., Paller C.J., Rosner I., Han M. (2019). PSA Doubling Time and Absolute PSA Predict Metastasis-free Survival in Men with Biochemically Recurrent Prostate Cancer After Radical Prostatectomy. Clin. Genitourin. Cancer.

[18] Freedland S.J., Rumble R.B., Finelli A., Chen R.C., Slovin S., Stein M.N., Mendelson D.S., Wackett C., Sandler H.M. (2014). Adjuvant and salvage radiotherapy after prostatectomy: American Society of Clinical Oncology clinical practice guideline endorsement. J. Clin. Oncol..

[19] Broeck T.V.D., Bergh R.C.V.D., Arfi N., Gross T., Moris L., Briers E., Cumberbatch M., De Santis M., Tilki D., Fanti S. (2019). Prognostic Value of Biochemical Recurrence Following Treatment with Curative Intent for Prostate Cancer: A Systematic Review. Eur. Urol..

[20] Feng M., Hanlon A.L., Pisansky T.M., Kuban D., Catton C.N., Michalski J.M., Zelefsky M.J., Kupelian P.A., Pollack A., Kestin L.L. (2007). Predictive factors for late genitourinary and gastrointestinal toxicity in patients with prostate cancer treated with adjuvant or salvage radiotherapy. Int. J. Radiat. Oncol. Biol. Phys..

