# Supplementary material for: Outcomes and Prediction Models for Exclusive Prostate Bed Salvage Radiotherapy among Patients with Biochemical Recurrence after Radical Prostatectomy
Source: Cancers (Basel). 2021 May 28;13(11):2672. doi: 10.3390/cancers13112672 (PMC8199341; doi:10.3390/cancers13112672)
Supplement: Supplementary file 1 [file cancers-13-02672-s001.zip › cancers-1202068-supplementary.pdf]

cancers

MDPI

Article

Outcomes and Prediction Models for Exclusive Prostate Bed Salvage Radiotherapy among Patients with Biochemical Recurrence after Radical Prostatectomy

Chi-Shin Tseng [1,2,3], Yu-Jen Wang [4,5], Chung-Hsin Chen [2], Shuo-Meng Wang [2], Kuo-How Huang [2], Po-Ming Chow [2], Yeong-Shiau Pu [2], Chao-Yuan Huang [2,*] and Jason Chia-Hsien Cheng [1,6,7,*]

1 Graduate Institute of Clinical Medicine, National Taiwan University College of Medicine, Taipei 100233, Taiwan; clifford1987tcs@gmail.com (C.-S.T.); jasoncheng@ntu.edu.tw (J.C.-H.C.)
2 Department of Urology, National Taiwan University College of Medicine and Hospital, Taipei 100225, Taiwan; mufasachen@gmail.com (C.-H.C.); dturo62smw@gmail.com (S.-M.W.); kuohowhuang@gmail.com (K.-H.H.); meow1812@gmail.com (P.-M.C.); yspu@ntu.edu.tw (Y.-S.P.)
3 Department of Medicine, National Taiwan University Hospital Jin-Shan Branch, New Taipei City 208204, Taiwan
4 School of Medicine, College of Medicine, Fu Jen Catholic University, New Taipei City 242062, Taiwan; yu-jen.wang@gmail.com (Y.-J.W.)
5 Department of Radiation Oncology and School of Medicine, Fu-Jen Catholic University Hospital and College of Medicine, New Taipei City 243089, Taiwan
6 Division of Radiation Oncology, Department of Oncology, National Taiwan University College of Medicine and Hospital, Taipei 100229, Taiwan
7 Graduate Institute of Oncology, National Taiwan University College of Medicine, Taipei 100233, Taiwan
* Correspondence: cyh540909@gmail.com (C.-Y.H.); jasoncheng@ntu.edu.tw (J.C.-H.C.); Tel.: +886-2356-2842 (J.C.-H.C.); Fax: +886-2331-2172 (J.C.-H.C.)

Citation: Tseng, C.-S.; Wang, Y.-J.; Chen, C.-H.; Wang, S.-M.; Huang, K.-H.; Chow, P.-M.; Pu, Y.-S.; Huang, C.-Y.; Cheng, J.C.-H. Outcomes and Prediction Models for Exclusive Prostate Bed Salvage Radiotherapy among Patients with Biochemical Recurrence after Radical Prostatectomy. *Cancers* **2021**, *13*, 2672. https://doi.org/10.3390/cancers13112672

Received: date
Accepted: date
Published: 28 May 2021

Publisher's Note: MDPI stays neutral with regard to jurisdictional claims in published maps and institutional affiliations.

Copyright: © 2021 by the authors. Licensee MDPI, Basel, Switzerland. This article is an open access article distributed under the terms and conditions of the Creative Commons Attribution (CC BY) license (http://creativecommons.org/licenses/by/4.0/).

Table S1. Biochemical recurrence (BCR), androgen deprivation therapy (ADT), and metastasis event numbers according to different combinations of the risk factors: Pre-salvage radiotherapy (SRT) PSA and PSA-doubling time (PSA-DT).

No. of Risk Factors	Pre-SRT PSA	PSA-DT	Case Numbers	BCR Events (%)	ADT Events (%)	Metastasis Events (%)
0	-	-	15	2 (13%)	0 (0%)	0 (0%)
1	-	+	35	14 (40%)	10 (29%)	6 (17%)
1	+	-	11	5 (45%)	2 (18%)	0 (0%)
2	+	+	30	19 (63%)	15 (50%)	11 (37%)

"-" for Pre-SRT PSA < 0.45 ng/mL or PSA-DT >8months
"+" for Pre-SRT PSA 0.45-1.5 ng/mL or PSA-DT ≤8 months
